# Strengthening the COVID-19 pandemic response, global leadership, and international cooperation through global health diplomacy

**DOI:** 10.34172/hpp.2020.48

**Published:** 2020-11-07

**Authors:** Sumbal Javed, Vijay Kumar Chattu

**Affiliations:** ^1^School of Public Health, The University of Hong Kong, Hong Kong; ^2^Department of Medicine, Faculty of Medicine, University of Toronto, Toronto, Canada; ^3^Institute of International Relations, The University of the West Indies, St. Augustine, Trinidad and Tobago

**Keywords:** COVID-19, Global health, Diplomacy, International cooperation, World Health Organization, Pandemic, International health regulations

## Abstract

The coronavirus disease 2019 (COVID-19) pandemic continues to claim lives around the world and, to some extent, reflects the failure of international cooperation. Global health diplomacy (GHD)can be a bridge for international cooperation for tackling public health crises, strengthening health systems through emphasizing universal health coverage for sustainable and equitable development, and rebuilding multilateral organizations. It can be a catalyst for future global health initiatives. Health should not be used as a political tool at the cost of people’s lives, nor should it become a proxy for geopolitics but can be used to diffuse tensions and create a positive environment for political dialogue. Health diplomacy’s focus should be to mitigate inequality by making available diagnostics, therapeutics, and vaccines as a global public good. The implications for the lack of international cooperation will lead to increased global disparities and inequities as the countries that cannot procure vaccines will find their population more vulnerable to the pandemic’s repercussion. Though the international cooperation on trade has suffered the impact of geopolitical shifts and competition, through engaging in GHD, the governments can align the trade and health policies. Amid this global health crisis, the World Health Organization (WHO) has faced an increase in International Health Regulations violations, limiting its influence and response during this COVID-19 pandemic. Nations need to develop a sense of cooperation that serves as the basis for a mutual strategic trust for international development. The priorities of all the countries should be to find the areas of common interest, common operational overlap on development issues, and resource allocation for this global fight against COVID-19.

## Introduction

The coronavirus disease 2019 (COVID-19, has affected millions with 44.8 million confirmed cases and claimed over 1.18 million lives as of October 30, 2020.^1^ While countries are grappling with how best to tackle this virus and its repercussion on health systems, societies, and economies, our scientific understanding of COVID-19 and the best public health measures to combat it, continues to progress. If the current pandemic has made anything clear again, health threats and challenges transcend national borders. As the disease is a natural part of our world, it was never a big question. Still, when the COVID-19 outbreak occurred, many countries initially struggled to respond adequately and lacked sufficient resources. The availability of scanty information about the new virus and lack of preparedness meant losing crucial time to devising instead of taking concrete actions against COVID-19. No single institution is at fault as it is a reflection of a collective international cooperation failure. As stated by the World Health Organization (WHO), global health diplomacy (GHD) connects the disciplines of public health, law, international relations, management, and economics, focusing on negotiations and influencing the global policy environment for health. The core principle of GHD is countries joining in the diplomatic fora to handle public health problems.^2^ The Contingency Fund for Emergencies of WHO gives only 24 hours to respond to disease epidemics and other disasters – which helps to contain health emergencies, thus effectively saving lives; however, it has been habitually underfunded.^3^ It is evident from the President of the United States to stop funding WHO that the growth of populist sentiment throughout the world is yet another blow to the multilateral system.^4^ The US President’s decision could not have been more ill-timed. It is truly a reflection of a nationalist agenda that views multilateral institutions and the very notion of global governance as its bête noire. As there are many geopolitical shifts, change in the world order with disturbed bilateral relations between major powers seen recently and more after the COVID-19 pandemic resulted in a lack of international cooperation in handling this pandemic. This perspective examines these trends in the past few months. It addresses the growing need for GHD as a discipline, practice at various regional/global platforms and its potential in strengthening health security and international cooperation. The findings are described below with evidence from the published literature and online web resources.

### Funding issues of the World Health Organization 

Every year, the WHO’s budget is financed through a mix of assessed and voluntary member states’ voluntary contributions. Countries need to pay assessed contributions to be a member of the Organization; assessed contributions remain a crucial source of financing for the Organization, minimizing dependence on a narrow donor base. It further allows resources to be aligned to the Program Budget.^5^ Interestingly, the assessed contributions have declined significantly as an overall percentage of the Program-Budget for many years, e.g., in 1971, 62% of assistance to the WHO came from the mandatory assessed contribution, which has declined now to approximately 18%. We must address this and rebuild the compulsory contribution^6^ and ensure active participation by each member state. Progress has been slow and, in some instances, agonizingly too late. Recently, the Director-General of WHO informed the Executive Board, “For too long, the world has operated on a cycle of panic and neglect. We throw money at an outbreak, and when it’s over, we forget about it and do nothing to prevent the next one…If we fail to prepare, we are preparing to fail.” ^7^

### Global health diplomacy as a catalyst for international cooperation 

It is time to acknowledge that the security of every nation in part relies on global health security. In the past years, many leaders have parted from the notion of global cooperation. Lack of International cooperation risks impeding a successful and integrated global approach towards handling COVID-19 worldwide. The Oslo Ministerial Declaration in 2007 accredited health as a highly important but disregarded foreign policy issue. Health as a forging policy issue on the international agenda requires attention and a robust strategy.^8^ Protecting and advancing public health as part of the foreign policy agenda is justifiable. However, the current COVID-19 pandemic, to some extent, reflects the failure of international cooperation. GHD can be a bridge for international cooperation for tackling public health crises, strengthening health systems through emphasizing universal health coverage for sustainable and equitable development, and rebuilding multilateral organizations. It can be a catalyst for future global health initiatives. However, health should not be used as a political tool at the cost of people’s lives, nor should it become a proxy for geopolitics but can be used to diffuse tensions and create a positive environment for political dialogue.

To advance the practice of GHD through capacity building programs, it is essential to have strong leadership and a long-term commitment to training both the cadres’ health and non-health on the emerging issues in global health. Health Attachés are the connection among governments on global health issues. They successfully build and maintain association on every level of GHD and are uniquely positioned to advise on applying diplomacy in foreign affairs and global health. They act as the chief advisers to the diplomatic workforce on public health issues between their host countries and their own country. By identifying common interest areas and resolving contention between multiple stakeholders, Attachés demand a particular cross-cultural, multidisciplinary, and diplomatic set of skills. They can effectively handle many health and foreign policy challenges, including public health crises such as Ebola and COVID-19.

### Developing a cadre of global health diplomats 

Health Attachés have a highly specialized practice and perspective on combining global health with foreign affairs, placing them ideally on the front lines of GHD. However, there is inadequate training and a lack of well-defined career pathways for Health Attachés. For an effective practice of GHD and to promote global health by aligning public health and foreign policy outcomes, it is critical to establish a straight-forward career route for capacity building. It is imperative to be trained in international affairs to fully understand how different health issues fit inside foreign policy goals and objectives.^9^ It is worth noting that Thailand has attempted to develop the capacity for engaging in GHD; even though the active development might be an exception, it does offer some lessons for other countries. The capacity developed in Thailand aided the member states of the South-East Asia Region to build and strengthen their health and related professionals’ capacity. The Thai Ministry of Public Health collaborated with the WHO Regional Office and participated in the training courses on global health organized by the ThaiHealth Global-Link Initiative Program, which covered topics discussed at the 63rd World Health Assembly. Such initiatives would further develop and expand a specialized cadre of core practitioners of GHD so that the Health Attachés can advance common global health objectives within nations through diplomacy in this contemporary world.^9,10^

### Reimagining the role of global health diplomacy 

The prime focus of health diplomacy should be to mitigate inequality by making diagnostics, therapeutics, and vaccination available to all as a global public good. The implications for the lack of international cooperation will lead to increased global inequalities as the countries which are unable to procure vaccines will find their population more vulnerable to the pandemic’s repercussion. One of the effective means to do this is through a strong coordinated body such as WHO. The WHO Global Influenza Surveillance and Response System’s (WHO GISRS’s) aim is a just, transparent, impartial, methodical, effective system, on an equal footing. This framework applies to any other influenza viruses that may have the human pandemic potential apart from H5N1 and share the benefits such as access to vaccines. In contrast, the Pandemic Influenza Preparedness Framework’s objective is to enhance pandemic influenza’s preparedness, response, and strengthen the WHO GISRS.^11^ Besides, GHD and science diplomacy are coming together through the international cooperation of scientific institutions, researchers, and innovators. Furthermore, it is critical at this juncture as we want to develop diagnostics, therapeutics, and vaccines; therefore, health diplomacy and science diplomacy will play a crucial role, particularly as some countries would prefer that certain scientific results initially should be available to themselves.

## Discussion

### Navigating trade-health nexus through global health diplomacy

As countries deploy several measurements for combating the pandemic, rising COVID-19 patients, the under-funded healthcare systems of low-income countries may face even more hardships to provide access and medical treatment to its citizens. The Trade-Related Aspects of Intellectual Property Rights (TRIPS Agreement) and Public Health as part of the Doha Declaration has stated key flexibilities to countries in Article 31. They include the right to grant compulsory licenses, under clause 5, “each [WTO] member has the right to grant compulsory licenses and the freedom to determine the grounds upon which such licenses are granted.” Furthermore, clause 5(c) of the TRIPS Agreement stated that “public health crises, including those relating to HIV/AIDS, tuberculosis, malaria and other epidemics,” can constitute “a national emergency or other circumstances of extreme urgency.” It provides nations with some degree of flexibility in managing the patents for pharmaceuticals, which are public goods, especially in situations of “national emergencies” and “other circumstances of extreme urgency.”^12-14^ Many lessons were learned in the past during the HIV/AIDS epidemic that can also be applied to the current COVID-19, for example, when International cooperation helped strengthen Thailand’s’ capacity to implement compulsory licenses and TRIPS flexibilities.^15^ Through international collaboration, governments, multilateral organizations, and pharmaceutical companies can play an important role by supporting low-income countries on mandatory licensing, and they should not prevent or retaliate against them during a pandemic for pursuing such public health measures.^16^ While international cooperation on trade issues has suffered due to the geopolitical competition and shifts, through GHD, the governments could improve the nexus between both the public health and trade policies to combat this pandemic. Governments of 31 countries have imposed import duties of 30 percent on the soap; the U.S. has imposed tariffs on imports from China, leading to a shortage of medical supplies. This illustrates a common ground between trade and health, which can be negotiated through GHD. Conducive trade policy will facilitate imports and exports and improve logistics, which will also enhance the immunization rate in lower-income countries. Besides, it was also suggested that there should be a mechanism to support the medical services trade reform and e-health services to enable the smooth flow of medical knowledge and skills.^16-18^

### The new COVID-19 lessons for global leaders and international cooperation

The global shortage of personal protective equipment (PPE) such as surgical masks, N95 masks, respirators, hand sanitizers, gloves, face shields, disposable gowns, etc. for frontline health care workers did not only represent an ethical challenge but also a significant barrier to pandemic preparedness. Ultimately, these shortages posed a significant risk to both national and global health security. The high rates of infections and deaths in Italy were partly because healthcare workers had inadequate PPE access.^19^ In emergencies like this pandemic, there is a great need for adequate production and equipment distribution, which is crucial to caring for patients. To achieve such rapid targets, the scope is beyond the health sector. It needs a strong political will, commitment, action, and mobilization at all levels from local to global through various public-private partnerships. Distributions in the global supply chain led to the shortage of PPE in the United States and other European nations. Globally, China was the producer of roughly half of the world’s face masks before this pandemic. But, when the infection started to spread in China, the masks’ exports came to a standstill. However, once China’s transmission slowed down and the infection started to spread globally, China resumed exports to other countries as a goodwill gesture. The United States was not a major beneficiary due to their existing bi-lateral differences. As seen from various initiatives, China is often shown to be determined by having interests in foreign policy rather than pursuing health equality or providing humanitarian services. As evident from the spread of COVID-19, the world has paid a high price when a government curbs the free flow of information, particularly risk communication from its health professionals. Few global experts opined a great need for China to be more transparent and have a technical approach and change from its bureaucratic and top-down approach. Further, enhanced data transparency could have potentially mitigated the current public health crisis.^20,21^

The International Health Regulations (IHRs) were adopted by the U.N. a decade ago, immediately after the severe acute respiratory syndrome (SARS), to expedite the international coordination during public health emergencies. Many countries’ nationalist response to COVID-19 has challenged global governance and has examined the global health system’s legal foundations. Although these regulations are legally binding, they have certain drawbacks, such as the absence of an enforcement mechanism. All the governments are compelled to notify WHO if any public health event may constitute a Public Health Emergency of International Concern (PHEIC). Previously, PHEIC has been deployed to control the global spread of various infectious diseases in the past, namely, Polio, H1N1, Ebola, Zika, and most recently, COVID-19.^22^ GHD is viewed as a compulsory tool in the practice of smart diplomacy. During the Ebola outbreak in 2014, the global response was an important illustration of the critical need for the timely and effective practice of GHD. Several panels and committees reviewed the WHO’s performance and concluded that there was a very long (five-month) delay in declaring a PHEIC. A lack of clear communication and coordination among the WHO member states regarding travel restrictions and bans indicated a violation of the IHRs. Moreover, the global community’s limited response efforts during the epidemic draw great attention to the importance of effective diplomacy in emergency health situations.^23-25^

### Global divide, geopolitical shifts, and challenges amid COVID-19

Whereas the US President has decided to cut ties with WHO for allegedly conspiring with China for concealing the extent of the COVID-19 crisis, the Chinese President has announced to contribute the US $2 billion over the next two years while also claiming that China had informed WHO and the rest of the world on time.^26^ It is evident that during the COVID-19 crisis, governments have prioritized national interests over international cooperation. During this global public health crisis, WHO has faced an increase in IHR violations, limiting its influence and response during the COVID-19 pandemic. The agency doesn’t have the authority to investigate epidemics within countries independently and, as previously stated, has no enforcement power. Hence, it relies on the cooperation of member states. The U.S. decision to cut funding for WHO will not solve this problem intrinsic to the IHRs.^27^ To meet future threats, it is essential to revise the global health law as it has been unable to alleviate the impact of the current COVID-19 pandemic. It is crucial that international legal amendments clarify states’ obligations while facilitating legal accountability and realizing global health security, which may require either fundamental revisions to the existing IHRs or develop a new international legally binding instrument to strengthen the mechanisms for effective global health governance. Solidarity of member states will enhance global health governance and guarantee that the WHO receives enough support financially and politically. This will further empower it to confront governments that do not comply with the recommendations that evidence-based and scientifically proven.^22,27^

Major players in global health need to work together instead of attacking each other for achieving the common targets of improving the overall health status of global citizens. In this globalized world, no country can isolate itself. There is a need for a forward-looking view, and change is essential as the road ahead will require alliance-building and safeguarding human rights as they have a significant impact on whether we can achieve health security at the national and global levels health security. Failure of international cooperation would prevent patients from getting the essential health services they need and jeopardize the health of frontline workers and the operation of the entire health care system globally. Nations need to develop and nurture cooperation practices, which serve as the core for mutual strategic trust. The priority for all countries should be to explore and find common resource allocation, common interest, and common operational overlap on development issues.

### Framing Health and strengthening health systems through Global Health Diplomacy

The U.N. Secretary-General, António Guterres, in response to the emergence of COVID-19, pleaded all combatants to suspend the violent conflicts in their regions and ensure peace. “There should be only one fight in our world today,” he declared, “our shared battle against COVID-19.”^28^ Countries in a conflict that suffer from unique challenges with severe humanitarian crisis have to struggle more to cope with the repercussions of the pandemic. These include 1) lack of access to health services hindered availability of medical goods and various collaborations for scientific research; 2) withdrawal of humanitarian and health workers (the “chilling effect”). These risks can have a destabilizing effect on the conflict-ridden countries and result in catastrophic impacts for countries in neighboring regions. This will further continue to bear the effect of an increased influx of refugees, placing the additional risk on their fragile healthcare systems, the unsurmountable strain on the economy, and the risk of a future resurgence of the virus.

Therefore to mitigate some of the worst impacts of the pandemic, there is a need to recognize and reemphasize that health can 1) offer a good entry point for dialogue as part of efforts to promote peace and global health security through GHD,^29^ 2) focus its sole purpose of serving the public interest and 3) be used as a medium in building trust and legitimacy. A “global knowledge network” that cuts across borders despite the crisis can be a building block in peacebuilding efforts and need to maintain their independence and principles. To preserve access to health care services, health workers and health infrastructure require protection. There is a need to regularly monitor healthcare issues, especially on the indirect consequences on people’s health due to war and conflict, with a special focus on the vulnerable population, i.e., children and women as caregivers. Further, advocate strongly with an emphasis on issues of human health to include in the agenda of the United Nations Peace Building Commission, in collaboration and cooperation with the WHO.^8^

The Report of WHO on the “International Meeting on Health in All Policies” (HiAP) in 2010 emphasized that HiAP aims to collaborate “across sectors to achieve common goals. It is a strategy to include health considerations in policy making across different sectors that influence health, such as transportation, agriculture, land use, housing, public safety, and education. Therefore, HiAP reaffirms public health’s essential role in addressing policy and structural factors affecting health, as articulated by the Ten Essential Public Health Services, and it has been promoted as an opportunity for the public health sector to engage a broader array of partners.”^30^ Even after a decade, the HiAP is not implemented in most countries, leading to having weaker or fragile healthcare systems, especially in low-income countries in Africa and Asia. There is a great need for GHD to further the health and implementation of these key policies through international cooperation. Through successful negotiations, funding the health systems (e.g., infrastructure, technology, and surveillance) of low-income countries through multilateral organizations can be achieved. Through successful GHD, the nations can be sensitized for having a strong political commitment for prioritizing health and well-being of their citizens on which the economy runs. This COVID-19 pandemic can be a catalyst to galvanize the global cooperation long called for. There is an immediate need for cooperation and collaboration, an understanding of shared responsibility, and the critical aspects such as transparency, accountability, trust, and fairness. Successful GHD will not only assist in achieving the disease-specific national goals but also the attainment of health-related Sustainable Developmental Goals and universal health coverage at the global level.^31^

## Conclusions

COVID-19 has transformed the foreign policy–health linkage. We are now more aware that investing in health is pivotal to both the nation’s economic growth and human development and that any threats to human health may further jeopardize the stability and security. The successful practice of GHD and investing for the development of a cadre to further the practice benefits the nations for effective negotiations with a win-win situation with healthy partnerships and cooperation. This pandemic has reaffirmed that disease outbreaks do not respect geographical borders or the status of development. They can be handled with sure success only if nations work together by focusing on the shared interests in global health as the rationale and prioritizing health and health security by governments in their national plans as well as for the international health development.

## Acknowledgments

The authors thank the reviewers for their critical feedback and for improving the quality of this manuscript.

## Funding

Nil.

## Competing interests

The authors declare that there is no conflict of interests.

## Ethical approval

**N**ot applicable.

## Authors’ contributions

The authors SJ and VKC were involved in concept, design, literature search, data acquisition, and manuscript preparation. VKC did the manuscript editing and manuscript final review. Both the authors SJ and VKC, have approved the final version of the manuscript.
